# Event-related potential alterations in fragile X syndrome

**DOI:** 10.3389/fnhum.2012.00264

**Published:** 2012-09-24

**Authors:** Inga S. Knoth, Sarah Lippé

**Affiliations:** ^1^Centre de Recherche CHU Ste-Justine, University of MontrealMontreal, QC, Canada; ^2^Centre de Recherche en Neuropsychologie et Cognition, University of MontrealMontreal, QC, Canada

**Keywords:** fragile X syndrome, event-related potential, cognition, intellectual disability, autism spectrum disorders

## Abstract

Fragile X Syndrome (FXS) is the most common form of X-linked intellectual disability (ID), associated with a wide range of cognitive and behavioral impairments. FXS is caused by a trinucleotide repeat expansion in the FMR1 gene located on the X-chromosome. FMR1 is expected to prevent the expression of the “fragile X mental retardation protein (FMRP)”, which results in altered structural and functional development of the synapse, including a loss of synaptic plasticity. This review aims to unveil the contribution of electrophysiological signal studies for the understanding of the information processing impairments in FXS patients. We discuss relevant event-related potential (ERP) studies conducted with full mutation FXS patients and clinical populations sharing symptoms with FXS in a developmental perspective. Specific deviances found in FXS ERP profiles are described. Alterations are reported in N1, P2, Mismatch Negativity (MMN), N2, and P3 components in FXS compared to healthy controls. Particularly, deviances in N1 and P2 amplitude seem to be specific to FXS. The presented results suggest a cascade of impaired information processes that are in line with symptoms and anatomical findings in FXS.

## Introduction

### Intellectual disability and Fragile X Syndrome

Intellectual disability (ID) is among the most common and severe handicaps of childhood. It is defined as “a condition of arrested or incomplete development of the mind, which is especially characterized by impairment of skills manifested during the developmental period, skills which contribute to the overall level of intelligence, i.e., cognitive, language, motor, and social abilities” (World Health Organization, 2004). Generally, Standard Intelligence Quotient (IQ) tests with a mean of 100 and a standard deviation of 15 are used for diagnosis. In this context, ID is determined by assessing an IQ <70 (i.e., less than 2 standard deviations below the mean) (Ropers, 2010). Numerous genetic and environmental factors can cause ID. They remain unknown in 30–50% of cases (Daily et al., 2000). Among genetic causes, X-linked recessive gene defects are believed to be responsible for approximately 10–12% of ID found in males (Ropers and Hamel, 2005). The most common form of X-linked mental retardation is the Fragile X Syndrome (FXS), which affects about 2% of male ID patients (Ropers and Hamel, 2005). FXS is caused by a trinucleotide repeat expansion in the FMR1 gene, which is located on the X-chromosome. Generally, it follows the hereditary transmission of X-chromosomal inheritance, but with some particular features. Firstly, despite their existing non-mutated X-chromosome, women can also be affected (approximately half of the prevalence found in men) but with greater variation in the phenotype expression (Bennetto et al., 2001). Besides the full mutation of more than 200 repeats which underlies FXS in comparison to the normal length of 30 triplets, there also exists a premutation with an intermediate length between 55 and 200 repeats. This premutation leads to non-penetrant carriers, who may pass on a full mutation to their child, due to the instability of the premutation in meiosis (Bassell and Warren, 2008). According to the mGluR theory of FXS, the FMR1 gene prevents expression of the encoded “fragile X mental retardation protein (FMRP)” (Bear et al., 2004). Normally, FMRP is known to repress the translation of specific mRNAs in response to the activation of metabotropic Glutamate Receptors (mGluRs). In turn, mGluRs are regulated by the inhibitory GABAergic system presynaptically, a putative altered mechanism in FXS. In Fragile X patients, the absence of FMRP leads to altered structural and functional development of the synapse. On the structural level, altered dendritic development, including increased density of dendritic spines, weak, elongated dendritic spines, and immature synaptic connections, are found in FXS patients and FXS animal models (Comery et al., 1997). Functionally, the FMRP deficit results in an exaggerated mRNA translation and thus causes continuous enhanced mGluR-dependent long-term depression. Consequently, the protein-synthesis in the synapses is not modified specifically to stimuli induction and therefore a loss of protein synthesis-dependent plasticity occurs (Bassell and Warren, 2008). The FMRP absence might therefore prevent activity-based synapse maturation and synaptic pruning, which is essential for normal brain development (Weiler and Greenough, 1999) and cognitive development (Schneider et al., 2009). In this context, the mGluR5 inhibitors were investigated as possible medical treatments for the FXS phenotype in several animal models (Krueger and Bear, 2011). Subsequent to the finding of a number of reversed phenotypes in animal models, clinical trials with human patients have been initiated and show promising preliminary results (Berry-Kravis et al., 2009).

In this review, we aim at unveiling the contribution of electrophysiological signal studies for the understanding of information processing impairments of a common intellectual deficiency syndrome, FXS.

### Cognitive impairments found in FXS

The ID in FXS does not globally extend to all cognitive domains, but concerns abilities within and across specific domains, which show stability into adulthood (Cornish et al., 2008). In most cases, vocabulary, verbal working memory and long-term memory for meaningful information are well preserved (Cornish et al., 2005), whereas the cognitive and behavioral domains listed in Table 1 tend to be affected frequently. Since the FXS phenotype shows great variability from case to case, the mentioned symptoms occur in some, but not all, FXS patients. In addition, the intensity of the symptoms ranges from mild to severe (Schneider et al., 2009). The deficits shown in behavior and social cognition, marked in gray within the table, are shared with disorders belonging to the autistic spectrum; about 30% of male individuals with FXS meet the diagnostic criteria for autism (Rogers et al., 2001).

**Table 1:** **Symptoms frequently found in FXS patients sorted by domains**.

**Domain**	**Symptoms frequently found in FXS patients**
Behavior	Pervasive hyperactivity and impulsivity (Bregman et al., 1988; Baumgardner et al., 1995; Schneider et al., 2009)
	Stereotyped behavior, self injury, perseverative preoccupations, and interest (Bregman et al., 1988)
	Poor fine and gross motor coordination (Loesch et al., 2003)
	Delayed socialization and avoidance (Budimirovic et al., 2006)
Social cognition	Gaze aversion (Bregman et al., 1988; Schneider et al., 2009)
	Impaired face recognition and emotion perception (Turk and Cornish, 1998)
	Theory of mind (Garner et al., 1999)
Language	Delayed echolalia (Bregman et al., 1988; Cornish et al., 2005; Schneider et al., 2009)
	Idiosyncratic responses (Bregman et al., 1988)
	Abnormalities in intonation and rhythm (Bregman et al., 1988)
	Verbal perseveration (Bregman et al., 1988; Schneider et al., 2009)
	Cluttering of speech (Cornish et al., 2005)
	Tangential language (Sudhalter and Belser, 2001)
Executive functions	Working memory (Cornish et al., 2005, 2008; Schneider et al., 2009)
	Planning and set shifting (Schneider et al., 2009)
	Deficits in attentional control (Bregman et al., 1988; Cornish et al., 2005)
	Inhibition (Cornish et al., 2005)
	Sequential processing (Loesch et al., 2003)
Emotional stability	Anxiety disorders (Bregman et al., 1988; Cornish et al., 2005; Schneider et al., 2009)
	Social avoidance (Cornish et al., 2005; Schneider et al., 2009)
	Aggression (Schneider et al., 2009)
Visual-spatial cognition	Impairments in visual-spatial reasoning (Cornish et al., 2008; Schneider et al., 2009)
	Object occlusion (Farzin and Rivera, 2010) Arithmetic problems (Loesch et al., 2003)
Hyperarousal	Hyperarousal to sensory stimuli (Schneider et al., 2009)

Although non-exhaustive, Table 1 shows a wide range of cognitive impairments in FXS patients. Most studies have investigated patients with FXS full mutation; however, it is worth mentioning that a recent study found attentionally based enumeration impairments in premutation carriers (Goodrich-Hunsaker et al., 2011). Premutation carriers may thus also present subtle cognitive impairments.

### ERP findings in FXS

In order to address maturational abnormalities in FXS, cortical and subcortical morphology have been studied and were found to be associated with alterations in cognition (Meguid et al., 2012). Given the availability of the Event Related Potential technique and its capacity to record local field potentials, which are summarized postsynaptic potentials from large groups of neurons (Luck, 2005), it is surprising that only a few ERP studies have addressed FXS, in which synaptic plasticity is assumed to be impaired. Indeed, five relevant ERP studies conducted with full mutation FXS patients have been published since the 1980s (St. Clair et al., 1987; Rojas et al., 2001; Castrèn et al., 2003; Van der Molen et al., 2012a, b). After a short description of the applied study designs, their findings will be presented in an order corresponding to the investigated ERP components.

### Study design

Table 2 shows the study population characteristics in the reviewed studies. Samples varied between 5 and 28 individuals, from children to adults, and male and female frequency varied between studies' samples^1^.

**Table 2:** **Study population characteristics in the reviewed studies**.

**Study**	**FXS patients**				**Healthy controls**			**Down syndrome**		
	***N***	**Age in years**	**Mental age/IQ**	**Co-morbidity**	***N***	**Age in years**	**IQ**	***N***	**Age in years**	**Mental age**
St. Clair et al., 1987	28, 2♀, 26♀	16–66 M: 43 (± 13)	1.8–4.6 M: 3.08 (± 0.08)	No epilepsy, no autism	83	18–75	N.A.	90	16–66	3 (± 1.5)
								36	16–37	2.17 (± 1.5)
								54	38–66	
Rojas et al., 2001	11, 6♀, 5♂	M: 28.95 (± 2.51)	IQ: 67.55 (± 5.47)	N.A.	11, 6♀, 5♂	M: 28.83 (± 2.51)	127.5 (± 2.9)			
Castrèn et al., 2003	5♂	7–13 M: 11.6 (± 2.8) 1 × 28	N.A.	No epileptic seizures, no medication	4♂	M: 10.6 (± 0.6)	N.A.			
Van der Molen et al., 2012a, b	16/11♂	18–42 M: 29.6	7.7 (± 1.6)	No medication	20/22♂	19–47 M: 29.2	121.5 (± 25.8)

All researchers investigated full mutation FXS patients and age-matched healthy controls. However, St. Clair and colleagues included an additional control group with ID, i.e., Down syndrome (DS). This control group enabled differentiation between obtained effects that rely on the level of brain development and effects that are specific for brain mechanisms underlying FXS. Therefore, the developmental level of the FXS patients has to be considered as a confounding variable to the results of the other four studies. Both chronological and mental age show considerable variation among the reported studies, ranging from children in Castrèn's study to patients in retirement age in St. Clair's study. This variation has to be kept in mind when results between the studies are compared, since both chronological and developmental age is expected to influence ERP waves (Courchesne, 1990). The IQs reported for the control subjects in Rojas and Van der Molen's studies are strikingly high, which probably reflects the tendency to recruit controls in the university setting, since years of education are positively correlated with IQ (Rowe et al., 1998).

The higher prevalence of FXS full mutations in men is reflected in the gender distribution in the majority of the studies. By contrast, Rojas and colleagues investigated more female FXS patients (Rojas et al., 2001), which might account for the rather moderate ID found in their population compared to the other three studies which provide maturational age for their FXS patients (St. Clair et al., 1987; Van der Molen et al., 2012a,b), since the female FXS phenotype shows more variability (Bennetto et al., 2001).

The authors reported little on possible comorbidities in the investigated patients. Only St. Clair and colleagues specifically mentioned the absence of autism in their population (Primrose et al., 1986), whereas most of the other studies mainly controlled for epilepsy and medication. All participants were tested for sufficient hearing.

Experimental procedures used in the reviewed studies are listed in Table 3. All studies investigated the auditory modality. However, Van der Molen and colleagues (2012b) investigated the visual modality in their second study. Except for Rojas and colleagues (2001), all other studies made use of oddball paradigms—active in half of the cases and passive in the other half. St. Clair's group did not report the behavioral outcomes of their task, nor did they connect them with the recorded brainwaves, since they used it predominantly to check if the participants were able to perceive the difference between the standard and the deviant tone.

**Table 3:** **Comparison of the experimental procedures used in the reviewed studies**.

**Study**	**Experimental paradigm**	**Stimuli parameters**	**Task**
St. Clair et al., 1987	Active auditory oddball paradigm	Standard/Deviant tone (1000/1500 Hz)	Count aloud infrequent tones (check of task comprehension)
		Ratio 9:1	
		Stimulus rate 1.1/s	
		Intensity: 75 dB binaurally through headphones	
		Stimulus duration: 20 ms, rise/fall time 9.9 ms	
Rojas et al., 2001	Presentation of pure tones	1000 Hz sine-wave tone	No task, participants watched silent movie
		Intensity: 80 dB monaurally through headphones	
		Stimulus duration: 30 ms, rise/fall time 5 ms	
		4 s inter-stimulus interval	
Castrèn et al., 2003	Passive auditory oddball paradigm (only ERP to standard tones were analysed)	Standard/deviant tone (800/560 Hz)	No task, participants watched silent movie
		Ratio 8.5:1.5	
		Stimulus duration: 84 ms, rise/fall time 7 ms	
		Intensity: 60 dB above subject's hearing threshold, right ear through headphones	
		1s inter-stimulus-interval	
	Auditory habituation	Trains with four identical standard tones		
		1 s inter-stimulus interval	
		12 s inter-train interval	
Van der Molen et al., 2012a	Passive auditory oddball paradigm	1000/1500 Hz sinusoidal tone	No task, participants watched silent movie
		Deviant/standard order counterbalanced across subjects	
		Ratio 9:1	
		Stimulus duration: 75 ms, rise/fall time 5 ms	
		Intensity: 80 dB binaurally through headphones	
		1 s inter-stimulus Interval	
Van der Molen et al., 2012b (Task order counterbalanced across subjects)	Active auditory oddball paradigm	1000/1500 Hz sinusoidal tone	-Response as quickly/accurate as possible to onset of deviant stimuli by pressing space bar
		Deviant/standard order counterbalanced across subjects	
		Ratio 8:2	-Responses (hits/false alarms, reaction times) registered within a 100-1200 ms time window after stimulus onset
		Stimulus duration: 100 ms, rise/fall time 5 ms	
		Intensity: 80 dB binaurally through headphones	
		500 ms inter-stimulus Interval	
	Active visual oddball paradigm	Blue/yellow coloured smiley faces 9.34 cd/m^2^, width 3.66°, height 3.68°	
		Centrally presented against black background (2.19 cd/m^2^) on a 17-inch laptop screen, 70 cm distance to screen	

Rojas and colleagues (2001) used Magnetoencephalography (MEG) as opposed to EEG. Their study is nevertheless considered in this review, since MEG signals are expected to originate from the same neurophysiological processes as EEG and offer evoked field potentials equivalent to ERPs. The details of the conducted EEG/MEG recording and analysis in the reviewed studies are summarized in Table 4. Obviously, the time span between the first study reviewed in this article, published by St. Clair and colleagues in 1987, and the most recent studies by Van der Molen and colleagues (2012a,b) has an influence on the technical sophistication of EEG recording and analysis equipment. The number of recording electrodes has increased as well as the computational possibilities to remove artifacts. Moreover, St. Clair and colleagues did not report separate results according to standard and deviant tones, even though they claim to have analysed them separately.

**Table 4:** **EEG/MEG registration and analysis in the reviewed studies**.

**Study**	**Electrodes**	**Processing**	**Component analysis**
St. Clair et al., 1987	1 Ag/AgCl-electrode at Cz, earlobe electrode as reference	Separated average for standard/deviant tones	N1, P2, N2, P3 determined through two independent rater
		500 trials total	Latencies/amplitudes calculated separately for each FXS patient
Rojas et al., 2001	4D Neuroimaging Magnes I neuro-magnetometer system, 37 axially-wound, first-order gradiometers, right-handed	Signal averaged separately for each hemisphere to obtain averaged auditory evoked magnetic field	P50 m, N100 m, P200 m observed in auditory-evoked field data
	Cartesian coordinate system as reference	Min. 150 trials/ear	Source analysis
Castrèn et al., 2003	19 Ag/AgCl electrodes, 10–20 system, right mastoid electrode as reference	Signal averaged for standard tones	N1, N2 determined at the highest peak amplitude site (Fz)
			Global field power
Van der Molen et al., 2012a	EasyCap electrode cap with 28 Ag/AgCl ring electrodes, left and right mastoid electrode as linked references	Average: 895/99 resp. 892/99 (standard/deviant) trials in controls resp. FXS patients	N1, P2, MMN, N2b, P3a at F3, Fz, F4, FC1, FCz, FC2, C3, Cz, C4, P3, Pz, P4, O1, Oz, and O2
			Peak amplitude defined by the method of local peak amplitude measurement (Luck, 2005), relative to the pre-stimulus baseline
Van der Molen et al., 2012b		Average: Auditory task: 236/58 resp. 234/59 (standard/deviant) trials for controls resp. FXS	
		Visual task: 216/48 resp. 212/48 trials for controls resp. FXS	

### ERP components investigated

ERPs enable us to extract neural responses associated with specific sensory, cognitive, or motor events from the overall EEG (Luck, 2005). Currently, ERPs are believed to reflect cerebral local field potentials, which are summarized postsynaptic potentials from large groups of neurons (Luck, 2005). Whereas the ERP technique enables an excellent temporal solution of 1 ms or better under optimal conditions, the spatial solution has to be studied with caution since the voltage measured at an electrode always reflects the summarized contributions from several different ERP generator sources (Luck, 2005).

The reviewed studies compare ERP components between FXS patients and control groups. The term “ERP component” can either simply describe the positive and negative voltage deflections within an ERP waveform according to the order or latency-window in which they occur (Luck, 2005) or it can refer to underlying cerebral generator processes, which contribute to the polarity of the recorded voltage deflection (Näätänen and Picton, 1987). Usually, the early components are related to sensory events and thus differ among modality, whereas the later components (starting with N2) are expected to reflect more cognitive phenomena. The reviewed studies reported results regarding auditory N1 and N2 (St. Clair et al., 1987; Castrèn et al., 2003; Van der Molen et al., 2012a,b), auditory P2 and P3 (St. Clair et al., 1987; Van der Molen et al., 2012a,b) and auditory and visual MNN, visual N1, P2, N2, and P3 (Van der Molen et al., 2012b). This covers most of the commonly investigated auditory components and some of the cognitive components; however, it should be mentioned that other components exist, which might also allow interesting contributions to FXS research. Some of the predominantly cognitive ones will be addressed in the discussion toward the end of this article.

## N1

### Description of N1

The N1 is usually not the first major sensory response. In the auditory modality, brainstem evoked responses occur within the first 10 ms after stimulus onset, which are followed by midlatency components at around 10–50 ms and finally an auditory P1 at about 50 ms before the auditory N1 (Luck, 2005). In the visual modality, the first ERP component, the C1 wave, typically arises 40–60 ms after stimulus onset and shows a positive or negative deflection depending on which part of the visual field the stimulus is presented in (Luck, 2005). So far, no study has investigated the very early sensory components in FXS patients. Nevertheless, the main purpose of studying N1 in FXS is detecting alterations in early sensory stimulus processing. The auditory N1 peaks fronto-central at around 100 ms after the onset of an auditory stimulus, whereas the visual N1 peaks 30–40 ms later, at about 135 ms after the onset of a visual stimulus (Näätänen and Picton, 1987). Näätänen and Picton (1987) conclude in their review that the auditory N1 consists of three “true” components upon which three other stimulus-dependent components overlap. The first subcomponent is supposed to be a frontocentral negativity generated in the auditory cortex on the superior part of the temporal lobe. The second subcomponent, the T-complex, which peaks at temporal sites and consists of a positive wave at around 100 ms and a negative wave at 150 ms, probably stems from the auditory association cortices in the superior temporal gyrus. Lastly, there is a subcomponent of unknown source, generating a negative wave at the vertex at around 100 ms after stimulus onset, which is believed to reflect an unspecific reaction to sensory stimulation and often overlaps with the first described subcomponent.

The visual N1 was decomposed by Di Russo et al. (2002) into four subcomponents to find pairs of generator dipoles which fit the N1 complex. They suggest an occipital source for the early N150, which peaks at occipito–parietal sites and has a centro-parietal source for the fronto-central N155. The later temporo-parietal N180 and occipito-parietal N200 are expected to be associated with the early P1 sources in the lateral extrastriate cortex and the late P1 source in the ventral occipito–temporal cortex (Di Russo et al., 2002). Research interest has been focused on the effects of spatial attention (Luck et al., 2000) and discrimination processing (Vogel and Luck, 2000).

### N1 findings in FXS

St. Clair and colleagues (1987) reported that N1 latency in FXS did not differ from that in healthy controls, whereas it has been found to be significantly longer in patients with DS, during the active auditory oddball paradigm. N1 amplitude was found to be generally enhanced at vertex electrode Cz in response to both standard and deviant tones in FXS patients, compared to patients with DS and healthy controls. Rojas et al. (2001) considered the N1 equivalent in MEG, the N100 m auditory-evoked field potential, in response to pure tones and also found a significantly higher amplitude in FXS patients than in healthy aged matched controls. They further observed a difference in the lateralization of the N100 m source. While healthy adults show N100 m source location asymmetry (right anterior to left), a reduction in lateralization is found in FXS patients. The authors proposed that the reduced asymmetry either reflects a non-specific neurodevelopmental disturbance which occurs during prenatal development of cerebral asymmetry, since the phenomenon has also been found in schizophrenia (Reite et al., 1989, 1997), or stems from postnatal influences of the FXS mutation on the temporal lobe (Reiss et al., 1994). In either case, reduced N100 m source location asymmetry would be an outcome of disrupted brain development. Castrén and colleagues (2003) also found significantly larger auditory N1 amplitudes in FXS patients compared to healthy age matched controls in response to standard tones in their auditory oddball paradigm. This difference in N1 amplitude was most prominent in the frontal site Fz and was confirmed through global field power analysis. Van der Molen and colleagues (2012a) did not find any group differences of N1 latency at FCz. As for amplitude, they reported a significantly larger N1 amplitude to standard tones in FXS in a passive auditory oddball paradigm. This difference could be observed at electrodes Fz, the fronto-central FCz and Cz, whereas no differences were found for posterior sites. Further, the N1 amplitudes in controls were significantly larger for deviant than for standard tones, a difference which could not be found in FXS. Using an active oddball paradigm, a second study of Van der Molen et al. (2012b) again did not find any differences in N1 latency, neither in the auditory, nor in the visual modality. In the active auditory oddball paradigm, they reported larger N1 amplitudes for standard and deviant tones in FXS. In the active visual oddball paradigm, they found N1 peak amplitudes to be maximal at occipito-central electrode Oz in controls, but at FCz in FXS patients. At FCz the visual N1 amplitude was significantly larger for both stimuli in FXS than in controls. In both groups, visual N1 amplitude was larger at FCz than at Oz.

In addition, two groups tested habituation of N1 in response to stimulus repetition (Castrèn et al., 2003; Van der Molen et al., 2012a). Castrèn and colleagues (2003) tested short-term habituation of N1 to trains of four identical standard tones. Van der Molen and colleagues compared N1 response to late standard tones with N1 response to early standards. In both studies, controls showed a reduction of N1 amplitude after several presentations of the same tone, whereas no N1 habituation could be found in FXS patients.

Regarding behavioral results, Van der Molen's group (Van der Molen et al., 2012b) reported less accuracy, more false alarms and an increase in reaction time in FXS patients in both auditory and visual task compared to controls.

Summarizing the N1 findings in FXS, no differences in N1 latency were found, whereas all studies reported larger N1 amplitudes and a lack of N1 amplitude habituation in FXS compared to controls.

### Maturation of N1

The complexity of data concerning maturational changes of N1 makes it difficult to determine if the results obtained in FXS are due to brain alterations specifically underlying FXS, or if they are, at least partially, a phenomenon of delayed brain maturation. Moreover, whereas the auditory N1 characteristics are known to change with brain maturation, studies investigating these changes obtained inconsistent results (Mueller et al., 2008). Already the time point from which an N1 response can be consistently evoked is a matter of controversy. While some researchers found a clear N1 response in children at the age of 9 (Ruhnau et al., 2011), others only obtained a visible N1 in 9-year olds by filtering out slow activity (Ceponiene et al., 2002). In children younger than 9, some researchers managed to evoke an N1 response with longer inter-stimulus-intervals (Paetau et al., 1995), but others could not identify it reliably before 5 years of age (Lippé et al., 2009). The difficulties in detecting an N1 component in children might be due to an overlap of slow P1 and N2 waves, and also to a refractoriness of N1 generators in toddlers, which decreases with age (Ceponiene et al., 2002). According to peak location, the auditory N1 was found at temporal sites in children under six (Bruneau et al., 1997) and thereupon shifted to central sites (Tonnquist-Uhlen et al., 1995), as is prominently found in adults. The results regarding auditory N1 latency are more uniform, indicating a general decrease in latency with maturation (Ladish and Polich, 1989; Gomes et al., 2001; Ceponiene et al., 2002). The visual N1 shows a U-shaped pattern in amplitude from one month to 5 years of age (Lippe et al., 2007), and then a fairly uniform decrease in amplitude (Johnson, 1989; Brecelj et al., 2002) and latency (Johnson, 1989; Lippe et al., 2007). Finally, results concerning N1 amplitude again are somehow inconsistent. Whereas some researchers found an increase in auditory N1 amplitude from 5 to 19 years (Ladish and Polich, 1989), others found an N1 decrease for target tones from 8 to 17 years (Johnstone et al., 1996), while again others could not find any differences in auditory N1 amplitude from one month to 5 years of age (Lippé et al., 2009) nor from 7 to 20 (Johnson, 1989). Gomes and colleagues (Gomes et al., 2001) explained this inconsistency regarding auditory N1 amplitude by appeal to differences in maturation of the N1 subcomponents described above. They found no auditory N1 amplitude differences across age in what they call the central N1, which corresponds to the frontocentral N1 subcomponent described above. On the contrary, they found a lateral N1 amplitude decrease from childhood to adulthood at temporal electrodes, which corresponds to the T-complex subcomponent. This explanation is similar to Ceponiene and colleagues' account that proposed differently weighted N1 subcomponents in children and adults (Ceponiene et al., 2002).

With such controversy in N1 amplitude developmental characteristics, it is not appropriate to conclude of a delay of maturation in FXS. In fact, larger amplitude and the absence of differences in latencies do not fit the early developmental pattern of N1.

### N1 in ID and autism

Since N1 maturation results are mixed, they should be considered in other clinical populations that share some of the symptoms with FXS in order to determine if the results obtained in FXS are a more general phenomenon or if they are specific to it. Patients with ID show relatively consistently prolonged auditory N1 latencies in comparison with healthy controls. This was found by Yamamori et al. (2002) in 30% of young ID patients (1–19 years) in response to randomly presented fixed and enlarged tones. Similary, Ikeada et al. (2009) found longer N1 latencies in response to simple tones in a passive auditory oddball paradigm in their adult cultural-familial type and organic (no chromosomal abnormalities) ID patients. Prolonged N1 latencies in response to an active auditory oddball paradigm have also been found in adolescents (Seidl et al., 1997) and young adults (César et al., 2010) with DS. Using a visual active discrimination task, Henderson et al. (2000) observed prolonged N1 latencies in children with phenylketonuria. These results fit well with the developmental changes of N1 latency reduction described above, suggesting that prolonged N1 latency in comparison to age-matched controls displays retardation in the development of early sensory processing. It is therefore surprising that none of the studies investigating ERP in FXS found a prolonged N1 latency, while several other forms of ID show this characteristic. We might assume differences in the cerebral perturbations underlying some forms of ID and FXS. Results obtained in patients with autism and ID again shows a different picture. Ferri et al. (2003) investigated ERPs in subjects diagnosed with low-functioning autism and found significantly shorter N1 latencies in response to standard tones in a passive auditory oddball paradigm. The finding of normal N1 latency in FXS, whereas latency is prolonged in ID and shortened in autism, might offer a possibility to differentiate between these disorders even if we cannot yet concretely determine the underlying cerebral mechanisms.

The increase in N1 amplitude found in FXS might also be somehow specific for FXS, since the studies investigating N1 in ID (Yamamori et al., 2002; Ikeda et al., 2009), DS (Seidl et al., 1997; César et al., 2010), and phenylketonuria (Henderson et al., 2000) did not find differences in N1 amplitude between patient population and healthy controls. On the contrary, Henderson and colleagues found a smaller visual N1 in children with phenylketonuria in active discrimination tasks. Moyle et al. (2006) also found reduced visual N1 amplitudes in adults with phenylketonuria compared to healthy controls in a Go-Nogo task. In autism, the results concerning N1 amplitude are fairly inconsistent, which Bomba and colleagues (Bomba and Pang, 2004) explained in their review on auditory evoked potentials through the fact that older ERP studies did not take the developmental changes of N1 into account. More recent studies found either a reduced auditory N1 in response to randomly presented tones of varying intensity in autistic pre-school children with ID, compared to children only diagnosed with ID and healthy controls (Bruneau et al., 1999), or no difference between auditory N1 in response to a passive oddball paradigm in children and adolescents with low-functioning autism and healthy controls (Ferri et al., 2003).

### Hyperarousal in FXS as possible factor influencing N1

The auditory N1 complex is supposed to be determined by physical characteristics of the stimulus, such as onset, intensity, frequency, threshold, stimulus rate, and ear of stimulation. Similarly, the appearance of the visual N1 complex is influenced by physical stimulus characteristics, such as luminance (Johannes et al., 1995). Since physical characteristics of the stimulus are always held constant between clinical population and control group, it is unlikely that they are responsible for the increased N1 amplitude found in FXS compared to control groups (St. Clair et al., 1987; Rojas et al., 2001; Castrèn et al., 2003; Van der Molen et al., 2012a,b). However, the auditory N1 is known to be sensitive to subject factors, states of arousal, and level of performance (Näätänen and Picton, 1987). Näätänen and Picton reported several studies that found an increase in auditory N1 amplitude with higher levels of arousal and alertness. Considering the hyperarousal to sensory stimulation frequently found in FXS (Schneider et al., 2009), it seems probable that this generally higher state of arousal is reflected in an increased N1 amplitude in FXS.

The positive association between levels of performance and N1 amplitude (Näätänen and Picton, 1987) should be closely examined in this context. However, the only study reporting behavioral results and N1 characteristics in FXS is the second study by Van der Molen et al. (2012b), indicating that controls outperformed FXS patients on all behavioral measurements. Comparing the performance of FXS patients with healthy controls might not be appropriate to investigate this association. An ERP study comparing the N1 characteristics in a simple vs. a difficult task in FXS would therefore be interesting.

### Habituation of ERP components in ID and autism

Habituation of the N1 component, characterized by a decrease in N1 amplitude with stimulus repetition in controls (Karhu et al., 1997), is found to be attenuated in FXS. Habituation may be based on two mechanisms. First, the unspecific arousal response to the appearance of a new stimulus, which is part of the orienting reflex (Sokolov, 1963), is decreased after repetition (Karhu et al., 1997). Second, a strengthening of selective cortical connections occurs, which is expected to reflect the neural representation of the stimulus characteristics, and thus the memory trace. Surprisingly few studies investigated habituation of ERP components in populations with intellectual disabilities besides FXS and most of them are fairly old. Psatta (1981) investigated habituation of visual-evoked potentials in response to flashes in three groups of children with ID (idiopathic, exogenous, DS) and in age-matched healthy controls. In DS, which was the most impaired group, they found an inversed pattern of habituation, characterized through an increase in amplitude instead of a decrease. The other two groups with ID showed a reduction in amplitude in the later compared with earlier trials that, however, in contrast to the control group did not reach statistical significance. Thus, even though habituation of ERP components was visible in two groups with ID, it did not occur in the same extent as in normal controls. However, it should be kept in mind that Psatta compared the ID groups only to healthy individuals matched regarding their chronological, not their mental age. Schafer and Peeke (1982) found no habituation in auditory-evoked potentials in patients with DS in response to regularly presented clicks at electrode Cz, whereas healthy controls showed rapid habituation in the N1-P2-N2 complex. Karrer et al. (1995) investigated habituation of visual ERPs in DS using a passive oddball task with colored slides of two adult female faces serving as stimuli. They concluded that infants with DS habituate to repeated stimuli, indicated by a smaller N1 amplitude in response to frequent compared to novel trials. However, this habituation effect could only be found centrally (Cz), but not frontally (Fz). The authors explained this finding through either a different neural organization of visual discrimination in DS or a lack of habituation over the frontal cortex. As for autism, using a visual habituation and recovery paradigm, Verbaten et al. (1991) found no differences in decrease of negativity/positivity for N1, P1, N2, P3, N4, and P4 between autistic children without ID and healthy children, children with conduct disorder and children with emotional disorder. Thus, the autistic group showed no impairment in neuronal repetition suppression. A more recent study by Guiraud et al. (2011) investigated auditory habituation in infants at high-risk for autism (defined by having at least one full older sibling diagnosed with autism). They found poor habituation of P150 in response to standards in an auditory oddball paradigm in the high-risk, but not in the control group. The discrepancy between the two studies could be explained by differences between the visual and auditory modality in autism, whereas Courchesne et al. (1985) found less impairment in the processing of simple visual than auditory information in autism. However, studies targeting a high-risk group of infants should be treated with caution, since it is not clear if they will really develop an autistic spectrum disorder. Moreover, no information about the developmental stage of the subjects was given, which might have accounted more for the lack of habituation than a possible diagnosis of autism. With some qualifications, lack of neuronal habituation seems to be common in ID. Thus, the findings of an absence of N1 habituation in FXS support the hypothesis of neural adaptation being generally impaired in ID. Given that habituation is considered to be the most elementary form of learning, which occurs as early as the fetal stage (Morokuma et al., 2004), impairments in the underlying synaptic mechanisms may contribute to learning difficulties found in ID.

### Alterations in brain anatomy and deficient synaptic pruning in FXS as possible basis for deviances displayed in N1

On the neuronal level, this increased N1/N100 m amplitude in FXS patients suggests that more neurons are synchronously active in response to the stimulus presentation than in healthy controls (Rojas et al., 2001). Alternatively, sensory gain control mechanisms, which have been investigated in the context of selective attention (Hillyard et al., 1995), could account for the increased N1 amplitude. Gain control could be altered in FXS, in a way that signals get amplified constantly instead of only when stimuli are expected. These alterations might be related to either early, possibly even prenatal, alteration of neurodevelopment or delayed or otherwise disrupted synaptic pruning occurring postnatally. Comparing anatomic brain alterations that are found very early in FXS to cerebral alterations occuring later in life helps differentiate between these mechanisms (Hoeft et al., 2010). Volumetric, voxel-based, and surface-based modeling approaches in magnetic resonance imagery showed among other alterations a smaller superior temporal gyri in children and adults with FXS full mutation (Gothelf et al., 2008) compared to healthy subjects. In addition, greater gray matter volumes in occipito-temporal areas have been found in infants with FXS compared to normally developed and children with non-syndromic delay (Hoeft et al., 2010). As described above, these two regions are believed to be involved in auditory/visual N1 generation. Further, FXS toddlers showed a greater gray matter increase over time in temporal and occipital areas, which was interpreted as a possible indication of deficient synaptic pruning in FXS (Hoeft et al., 2007), fitting observations in animal models (Weiler and Greenough, 1999; Pfeiffer and Huber, 2007, 2009). According to these models, reduction of unnecessary neurons and synapses and strengthening of neuronal connections in order to compensate by tempting more efficient synaptic configurations are believed to be impaired in FXS.

However, assumptions regarding the underlying brain mechanisms remain hypothetical and should be addressed by combining EEG with other brain imaging techniques.

## P2

### Description of P2

Similar to N1, P2 is studied in FXS in order to reveal alterations in early sensory processing. The auditory P2 is the second ERP with positive polarity, occurring after N1 with a latency of approximately 50–250ms (Crowley and Colrain, 2004). Especially in older ERP-studies, the P2 was mainly referred to in combination with the N1 component, as the N1-P2 complex or “vertex potential,” but recent research suggested the potential of the P2 as a component on its own, which is the result of independent processes (Crowley and Colrain, 2004). In contrast to other components, the P2 has a similar scalp topography across auditory, somatosensory, and visual modalities, being maximal over the vertex (Crowley and Colrain, 2004). Previously, the auditory P2 sources were assumed to be located in the auditory cortex, but recent studies indicated more distributed sources, most likely in the mesencephalitic reticular activating system (Crowley and Colrain, 2004), the planum temporale, as well as the auditory association cortex (Godey et al., 2001). For the visual P2, source analyses suggested a generator in the parieto–occipital and temporal regions (Freunberger et al., 2007). Appearance of auditory P2 is influenced by stimulus characteristics like tone intensity, pitch, and inter-stimulus interval, as well as subject factors including attention and age (Crowley and Colrain, 2004). The visual P2 seems to be larger for animals than for non-animal nature scenes or simple visual patterns (Antal et al., 2000) and is also influenced by attention (Luck and Hillyard, 1994).

### P2 findings in FXS

St. Clair's group (St. Clair et al., 1987) reported no differences in auditory P2 latency between FXS patients, DS patients, and healthy controls. As for amplitude, they found the P2 amplitude to be significantly larger in FXS compared to DS and healthy controls. Rojas and colleagues did not investigate the P200 m responses, because they were only measureable in nine of 11 subjects in each group (Rojas et al., 2001). Similarly to St. Clair, Van der Molen and colleagues found no differences between FXS and controls concerning P2 latency. This was the case in the passive auditory oddball paradigm (Van der Molen et al., 2012a) as well as in the active auditory and visual oddball paradigms (Van der Molen et al., 2012b). However, the latency in the active auditory oddball paradigm was found to be significantly shorter in both groups following deviant stimuli, in comparison to standard stimuli. In the passive auditory oddball paradigm, the P2 amplitude following both standard and deviant stimuli was larger at all sites in FXS than in controls (Van der Molen et al., 2012b). In controls, P2 amplitude in response to deviant stimuli was significantly smaller than in response to standard stimuli. This difference in P2 amplitude according to the probability of the stimulus could not be found in FXS. In contrast to this finding, FXS patients showed smaller P2 amplitudes following deviant stimuli in the active auditory oddball paradigm, as did controls, and P2 amplitudes were not found to be larger in FXS (Van der Molen et al., 2012b). In the visual modality, there was neither a difference between amplitudes in FXS and controls, nor did the probability of the stimulus have an effect. Consequently, the obtained P2 results are somehow inconsistent with an increased P2 amplitude in FXS only in the auditory modality, once in an active (St. Clair et al., 1987) and once in a passive paradigm (Van der Molen et al., 2012a), whereas in another study no differences in the active paradigm were found (Van der Molen et al., 2012b). Moreover, the FXS patients showed a difference in P2 amplitude between standard and deviant stimuli, but only in the active auditory paradigm, whereas controls showed this difference also in the passive paradigm. The lack of differences between visual P2 in FXS patients and controls is not discussed by the authors, but could reflect modality differences in stimulus processing, suggesting that visual processing in FXS is less impaired than auditory processing. This would be in line with the modality differences found in P3 amplitude discussed below. The most investigated influence on P2 amplitude is attention, with a decrease in P2 amplitude in response to an increase in level of attentiveness (Crowley and Colrain, 2004). Both groups show this effect in the active auditory but not in the visual oddball paradigm, in such a way that the P2 amplitude is decreased in response to deviant tones which require a behavioral response. Additionally, the controls show this difference in the passive oddball paradigm. It is possible that the controls paid more attention to the deviant tones even though no response was required, whereas the FXS patients might have been distracted by the silent movie which they watched during the task. However, this is only a hypothesis, and factors influencing the P2 in FXS patients need further research since the results obtained so far do not allow clear conclusions.

### Maturation of P2

The auditory P2 becomes a clearly distinguishable wave at all central sites at about age 10. The maximum peak shifts from Pz in younger children to Fz and Cz in older children and adults (Ponton et al., 2000). Changes in auditory P2 latency with maturation were not found by Johnstone and colleagues in children from 8 to 17 years (Johnstone et al., 1996), neither by Ponton's group in subjects from 5 to 20 (Ponton et al., 2000) or Mueller's group in different age groups between 9 and 74 (Mueller et al., 2008). However, it seems as though auditory P2 latency decreases with age between one month and 5 years of age (Lippé et al., 2009) and increases with age in adulthood (Picton et al., 1984; Anderer et al., 1996). The results concerning auditory P2 amplitude are more controversial. Johnstone and colleagues reported a P2 amplitude increase from 8 to 17 (Johnstone et al., 1996) and Mueller and colleagues found greater P2 amplitudes in the adult than in the child population (Mueller et al., 2008). Conversely, Ponton's group (Ponton et al., 2000) and Lippé's group (Lippé et al., 2009) observed a decrease in P2 amplitude across age. This controversy makes it difficult to determine if the alterations found in P2 amplitude in FXS might be caused by developmental delay.

### P2 in ID and autism

Three of the reviewed investigators who studied N1 reported similarly prolonged auditory P2 latencies in subjects with ID (Yamamori et al., 2002) and DS (Seidl et al., 1997; César et al., 2010), whereas no differences in amplitude were found. In one of the few studies mentioning P2 results, Lincoln and colleagues report no differences between subjects with autism, subjects with receptive developmental language disorder and healthy controls regarding P2 amplitude or latency in response to randomly presented tones differing in frequency and intensity (Lincoln et al., 1995). Thus, the increased auditory P2 amplitude partially found in FXS might be specific to FXS, since it is not found in other forms of ID or autism, whereas the prolonged P2 latency commonly found in ID is not observed in FXS. Congenital and developmental aberrations in the temporal lobe that affect areas believed to be involved in P2 generation (Gothelf et al., 2008; Hoeft et al., 2010) might be related to the P2 amplitude alterations found in FXS, as discussed for N1 above. Van der Molen and colleagues emphasize the influence that these early sensory processing deficits probably have on the generation of memory templates required for stimulus discrimination (Van der Molen et al., 2012a).

## Mismatch negativity—MMN

### Description of MMN

In contrast to the components discussed so far, which predominantly reflect early sensory processing, the MMN is the first cognitive component. The MMN has mainly been investigated in the auditory modality and describes a negative-deflecting wave that peaks maximally at central midline scalp sites between 160 and 220 ms in response to a mismatching stimulus occurring in a repetitive train of identical stimuli (Luck, 2005). Thus, the MMN reflects the brain mechanisms underlying the classification and differentiation of perceived stimuli. Two approaches explain the generation of MMN differently. Some authors describe the MMN as the outcome of a relatively automatic process not specifically requiring attention to compare incoming stimuli with a sensory memory trace of preceding stimuli (Alho, 1995). Sources are believed to lie in the auditory cortex, differing with stimulus characteristics, with supplementary sources in the frontal lobe and possibly in the hippocampus and the thalamus (Alho, 1995). In this approach, the MMN is seen as a process independent from the N1 with distinct source generators in the auditory cortex (Korzyukov et al., 1999). Other authors presented evidence suggesting a competing theory, namely, that MMN is not generated by separate auditory cortex sources, but rather arises from stimulus-specific adaptation of N1 activity (Jääskeläinen et al., 2004).

### MMN findings in FXS

Castrèn and colleagues only analyzed the ERPs in response to the standard stimulus in their oddball paradigm and therefore could not report MMN results (Castrèn et al., 2003). Van der Molen and colleagues investigated the MMN with a classical passive auditory oddball paradigm (Van der Molen et al., 2012b). They found a trend for longer MMN latency in controls compared to FXS, which did not reach significance. MMN was found to maximally peak at Cz in controls and Fz in FXS patients, with significantly smaller amplitude in FXS at Cz, Pz and Oz.

### Maturation of MMN

Auditory MMN is considered a developmentally stable ERP component that is already present in preterm infants (Cheour-Luhtanen et al., 1996) and thus might reflect information processing mechanisms developing very early in ontogenesis (Csepe, 1995; Cheour et al., 2000; Mueller et al., 2008). However, differences between MMN in infants and adults have been reported, including a decrease in latency with age (Cheour et al., 2000; Shafer et al., 2000; Mueller et al., 2008). Concerning MMN amplitude, smaller amplitudes in infants than in school-aged children and adults have been found (Oades et al., 1997; Cheour et al., 2000). In contrast, general (Shafer et al., 2000) or local decreases in amplitude with age (Gomot et al., 2000; Mueller et al., 2008) have also been reported. Thus, it has been suggested that the frontal system matures earlier than the sensory-specific temporal system (Gomot et al., 2000). According to these results, it might be possible that the reduced MMN amplitude found in FXS does reflect some sort of delay in brain maturation, but it cannot be said with certainty since the results concerning MMN amplitude maturation are not clear cut.

### MMN in ID and autism

Attenuated MMN amplitudes are frequently found in ID. Ikeda and colleagues conducted three studies investigating MMN in adult subjects with ID, using a passive oddball paradigm with synthetic vowels and pure tones. An attenuated MMN amplitude to both kinds of stimuli was found in patients with ID in all three studies (Ikeda et al., 2000, 2004, 2009), whereas greater MMN latencies in ID were only observed in the first study (Ikeda et al., 2000). Holopainen's group also found attenuated MMN amplitudes in a passive oddball paradigm at the individual maximal electrode for children with ID and children with dysphasia in comparison to a group with healthy control children, but no differences in latency (Holopainen et al., 1998). Nakagawa and colleagues found a smaller MMN amplitude in adults with ID compared to healthy controls in a passive oddball paradigm with fixed inter-stimulus intervals, whereas in conditions with random inter-stimulus-intervals the ID patients did not show any MMN (Nakagawa et al., 2002). By contrast, children with low-functioning autism tend to show shorter MMN latencies (Gomot et al., 2002; Ferri et al., 2003) and higher MMN amplitudes in response to novel, but not to deviant stimuli in comparison with healthy controls (Ferri et al., 2003). Therefore, it seems that MMN amplitude is generally reduced in several forms of ID, including FXS, whereas other forms of MMN alterations are found in autistic subjects with ID. In line with Jääskeläinen's theory (Jääskeläinen et al., 2004), perturbations in brain mechanisms underlying N1 would also account for alterations in MMN appearance. This may gain some support through the fact that FXS patients also show alterations in N1 amplitude. However, if the MMN is a component on its own with distinct sources in the temporal lobe, congenital, and developmental aberrations in the temporal lobe, such as those mentioned above under N1 and P2 (Gothelf et al., 2008; Hoeft et al., 2010), might contribute to MMN alterations in FXS.

## N2

### Description of N2

As mentioned above, N2 is one of the first cognitive components that have been studied in FXS. Several components are identified in the N2 time range. Luck differentiates between three types of N2: first, a basic N2, probably consisting of different subcomponents, and elicited by a repetitive, non-target stimulus (Näätänen and Picton, 1986); second, the MMN evoked by deviant, but task-irrelevant stimuli (sometimes also referred to as N2a); and finally a N2b that responds to deviant target stimuli and thus is expected to reflect stimulus categorization processes (Luck, 2005). This section will primarily discuss this last type of N2, the N2b. For auditory deviant stimuli, the N2b is largest over central sites, whereas it is maximal at posterior sites for visual stimuli (Simson et al., 1977). However, it is not clear if auditory and visual N2b reflect homologous neural processes (Luck, 2005). Sources for auditory N2 in response to target and novel stimuli were suggested in the temporal lobe, in the narrow area of the auditory cortex close to N1 generators (Albrecht et al., 2000), more specifically in the superior/middle temporal gyrus (Kiehl et al., 2001). Visual N2 generators were suggested to lie in the inferior temporal cortex (Wijers et al., 1997).

### N2 findings in FXS

The extent to which the N2 results of St. Clair and colleagues can be interpreted is limited. Even though they stated that they have averaged responses to frequent and rare tones separately, they only reported general N2 results, and it is not clear for which kind of stimulus the average is shown. Moreover, they did not report any behavioral results obtained through their active oddball paradigm task, since they only used it to control for whether participants were able to perceive the difference between the stimuli. Nevertheless, they found significantly longer N2 latencies in FXS and DS patients relative to healthy controls, but no differences in N2 amplitude between the three groups (St. Clair et al., 1987). Van der Molen and colleagues found that N2b maximally peaks at Oz in controls and at FCz in FXS patients in their passive auditory oddball paradigm (Van der Molen et al., 2012a). N2b latency was found to be shorter in response to deviant tones in both groups. Moreover, N2b latency was found to be longer in FXS patients in response to both stimuli compared to controls. N2b amplitude was larger in FXS than in controls, but only in response to standard stimuli. In controls, N2b amplitude differed between deviant and standard stimuli, with larger amplitude in response to deviant stimuli. This probability-based difference was not found in FXS patients. In the auditory active oddball paradigm, N2b peaked at Fz in controls and at FCz in FXS patients (Van der Molen et al., 2012b). N2b latencies were shorter in response to deviant tones in both groups, whereas FXS patients showed generally longer latencies. Larger auditory N2b amplitudes were found in FXS in response to both kinds of stimuli. In the visual modality, N2b peaked at F3 in controls for standard stimuli and at Fz for deviant stimuli, whereas it peaked at F4 in FXS patients for both stimuli. In controls, N2b latencies were shorter in response to deviants than to standards, whereas FXS patients tended to show an inversed pattern. FXS patients showed longer N2b latencies and larger N2b amplitudes than controls. In the active auditory and visual oddball paradigms, FXS patients were generally less accurate, slower, and showed more false alarms than the control group, and they committed significantly more false alarms in the auditory compared to the visual task (Van der Molen et al., 2012b). However, ERP results were not presented separately for correct vs. incorrect answers.

### Controversy in N2 results obtained in FXS

It is worth mentioning again that Van der Molen's group did not find differences in N2b amplitude in the active oddball paradigms. This absence of result is puzzling, since the N2b is supposed to be larger in response to task-relevant deviants. On the other hand, this larger N2b amplitude for deviants is observed in the passive oddball paradigm, which is normally known to elicit MMN rather than N2b. In the P2 section, it has been discussed that controls might have paid more attention to the deviant stimuli in the passive auditory paradigm, even though there were no task requirements, which could also account for the differences in N2b amplitude found in the passive oddball paradigm. Since the authors did not address this topic, it is difficult to determine which part of the paradigm might account for the missing differences between N2b amplitude in response to standards and deviants in the active paradigms. Further, this makes it more difficult to interpret alterations observed in FXS patients. Since St. Clair and colleagues also found the enhanced N2 amplitude, it could be a general phenomenon in FXS. On the other hand, Castrèn's group (Castrèn et al., 2003) found smaller N2 amplitudes in response to standard tones in FXS, which is in contrast to St. Clair and Van der Molen's findings. However, unlike St. Clair and Van der Molen's groups, Castrèn and colleagues investigated children with FXS and not adults. Further, they used a passive oddball paradigm and only report ERPs in response to standard tones. Thus, their N2 is most likely a basic N2, whereas the active paradigms of St. Clair and Van der Molen might also evoke N2b responses. St. Clair's group also found a more frontal N2 scalp distribution in patients with FXS compared to healthy controls, which is not reported in Van der Molen's active paradigms (Van der Molen et al., 2012b), but in the passive auditory paradigm with N2b peaking at Fz in FXS and Oz in controls (Van der Molen et al., 2012a). Thus, it seems that the more frontal distribution in FXS mainly appears in passive paradigms. The data for N2 in FXS is more controversial than for other components discussed so far, which might be partially due to the fact that it is more sensitive to changes in task parameters, making it more difficult to compare studies with different experimental designs. Supplementary differences between the study designs, which might have contributed to the inconsistent results, can be consulted in Tables 2, 3, and 4.

### Maturation of N2

In their review, Patel and Azzam suggest a maturation effect on N2b latency, which decreases with age and is directly associated with decreasing reaction times (Patel and Azzam, 2005). On the contrary, Mueller's group did not find differences in N2 latency between different age groups (Mueller et al., 2008). As for amplitude, a decrease in auditory N2 amplitude across age is reported (Johnstone et al., 1996; Mueller et al., 2008). Maximal N2 peak amplitude is known to move from posterior sites in infants to frontal sites in adults, beginning at approximately 14 years of age (Oades et al., 1997). Thus, the more frontal distribution of N2 in FXS children compared to controls found in passive paradigms by Castrèn's and Van der Molen's group is puzzling and cannot be explained through a delay in brain maturation. Only in Van der Molen's active auditory oddball paradigm do FXS patients show a more parietal N2b peak than controls, which would be in line with a delayed development of topography.

### N2 in ID

Findings in ID fit largely with the observed decrease in N2 latency with maturation, in a way that patients with ID (Yamamori et al., 2002) and DS (Seidl et al., 1997; César et al., 2010) showed prolonged N2 latencies compared with healthy controls, and thus showed a delayed maturation of N2. This is also supported by the fact that, in patients with DS, St. Clair and colleagues found the same N2 latency prolongation as in FXS (St. Clair et al., 1987). Thus, it seems as if the prolonged N2 latency in FXS is indeed a general phenomenon in ID, reflecting delayed brain maturation. Alterations in N2 amplitude were only reported by César et al. (2010), who found smaller N2 amplitudes in patients with DS. N2 amplitude findings are not only controversial in FXS, but also in other forms of ID, as is also the case for observations regarding the effect of brain maturation on N2 amplitude. Further research investigating N2 in well-controlled paradigms is therefore needed.

## P3

### Description of P3

Similar to N2, the components in the P3 time range of about 250–500 ms after stimulus onset can be broken down to several distinguishable ERPs. The main distinction is made between a frontal-central maximal P3a and a parietal maximal P3b component (Squires et al., 1975), which occur after unpredictable, infrequent deviances in stimulus characteristics. The P3a component is believed to be somewhat more automatic (Squires et al., 1975) and is elicited by truly unexpected or surprising stimuli (Verleger et al., 1994). The literature focuses mainly on the P3b component, which is often simply referred to as P3. The P3b occurs in response to task-relevant shifts and is sensitive to target probability, not to physical stimuli characteristics (Picton, 1992). The P3b is generated after the stimulus categorization process, but before response selection and execution (Luck, 1998). P3b amplitude increases in proportion to the effort devoted to the task (Isreal et al., 1980), but is also decreased by task difficulty (Luck, 1998), which complicates the interpretation of the component. There is no consensus in the field about the cognitive process reflected by P3b, but one frequently discussed hypothesis is the “context updating” process suggested by Donchin (1981), according to which the P3b reflects the updating of one's representation of the environment. In Polich's theoretical framework, P3a reflects focal attention processing which facilitates context maintenance, which itself is reflected by P3b and involves working memory operations. P3 is believed to be generated through frontal and temporal/parietal brain activation, suggesting a circuit pathway between these areas (Polich, 2007). The P3 is of particular interest in FXS, since it is known to be strongly determined by genetic factors and biological determinants (Polich, 2007).

### P3 findings in FXS

St. Clair and colleagues found a longer P3 latency in FXS and DS patients in comparison to the healthy control group (St. Clair et al., 1987). Additionally, the P3 amplitude in FXS and DS was found to be significantly smaller than in healthy controls. This was consistently found in FXS patients, independently of variables such as age, percentage of fragility, and intellectual functioning. St. Clair and colleagues found the P3 to be split in different components in some of their FXS patients. Seven out of 28 FXS patients showed a P3 clearly separated into two parts and several others showed partial P3 separation. It is not clear if the separation simply goes back to the P3a and P3b components or if it is caused by a genetic factor determining the ERP profile. They explored the relation between physical dysmorphysm, i.e., facial and testicular abnormalities, and complete separation of P3, since subjects without physical dysmorphism never showed completely separated P3 components. However, the correlation failed to reach significance. Another striking feature of the waveforms was that most of the FXS and some of the DS patients generated P3 in response to both frequent and infrequent stimuli. This lack of differentiation in P3 amplitude could not be traced back to an insufficient comprehension of the two-tone discrimination task, since the 28 patients chosen for analysis were all able to identify the deviant tones.

Van der Molen and colleagues (Van der Molen et al., 2012a,b) did not report an FXS specific separation of the P3 component, but, in contrast to St. Clair and colleagues, they investigated the P3a (Van der Molen et al., 2012a) and P3b (Van der Molen et al., 2012b) component separately, since they used a passive oddball paradigm in the first and an active oddball paradigm in the second study. Van der Molen's group found a prolonged P3a latency in FXS in response to standard and deviant tones in the passive auditory oddball paradigm. They also found differences in lateralization of P3a generation. Whereas the peak amplitudes for P3a were observed at the central midline in controls, the P3a peaked maximally at the left central electrode leads in FXS patients. Controls and FXS patients both showed larger P3a amplitudes in response to deviant in comparison to standard tones, whereas the amplitudes in response to deviant tones were larger in controls than in FXS. In the active auditory oddball paradigm, P3b latency was longer at Cz than at Oz in both groups. Additionally, P3b latency was longer in FXS patients than in controls at Cz. P3b amplitude peaked at Cz in controls for both kinds of stimuli, whereas it peaked at Oz in FXS for standard stimuli, and at Pz for deviant stimuli. FXS patients showed smaller P3b amplitudes in comparison to controls for standard and deviant tones. In the visual modality, FXS patients also showed longer P3b latencies than controls, which was significant for standard stimuli. Visual P3b peaked at Cz (standard stimuli) and Pz (deviant stimuli) in controls, and FCz (standard stimuli) and Oz (deviant stimuli) in FXS. Similar to the auditory conditions, visual P3b amplitude was significantly smaller in FXS in response to both stimuli, but was generally larger in response to deviant stimuli in both groups. Van der Molen and colleagues also found an interesting modality specific difference in FXS patients: P3b amplitude to auditory stimuli was significantly reduced in comparison to visual stimuli. The behavioral results matched the modality differences found in P3b amplitude, showing that FXS patients made fewer errors in the visual than in the auditory task. This difference was not found in controls. To assess if ERP components can predict behavioral performance, Van der Molen and colleagues carried out a regression analysis (Van der Molen et al., 2012b). The P3b amplitude relating to deviant auditory stimuli was the only ERP that could predict performance in the active oddball paradigm task. It predicted reaction time to deviant tones in FXS patients and controls, as well as the hit rate to deviant stimuli and the proportion of false alarms to standard stimuli in FXS patients. In the visual paradigm, this pattern could not be found, even though the P3b difference scores were considered to be the best explanation for the variance in reaction times to deviant stimuli in FXS patients. Since both MMN and P3a seem to be attenuated in FXS, the authors expected factors affecting the MMN component to also have an influence on the P3a component. However, linear regression analysis did not reveal a significant direct association between MMN and P3a latency or amplitude (Van der Molen et al., 2012a). To summarize, both groups investigating P3 in FXS found prolonged P3 latencies in active and passive auditory and active visual oddball paradigms (St. Clair et al., 1987; Van der Molen et al., 2012a,b). The most striking difference were the deviances in P3 amplitude observed in FXS. Even though Van der Molen and colleagues reported larger P3a and P3b amplitudes for deviant stimuli compared to standard stimuli in both controls and FXS patients (Van der Molen et al., 2012a,b), FXS patients still showed significantly reduced P3b amplitudes in both modalities.

Given that FXS patients already showed delays in N2, it is not surprising that P3 latency is also prolonged in comparison to controls. Moreover, P3 latency is known to be proportional to stimulus evaluating time and varies with individual differences in cognitive capability (Polich, 2007).

### Maturation of P3—P3 in ID and autism

Results concerning auditory P3 maturation consistently show a decrease in latency (Goodin et al., 1978; Johnson, 1989; Ladish and Polich, 1989; Pearce et al., 1989; Fuchigami et al., 1993; Johnstone et al., 1996) and an increase in amplitude (Ladish and Polich, 1989; Johnstone et al., 1996; Mueller et al., 2008) from childhood to adolescence. The same pattern is found in visual P3 maturation (Pfueller et al., 2011). Studies investigating P3 in ID are predominantly in line with these findings, suggesting a delayed maturation of the P3 component. Ikeda and colleagues found a decrease in auditory P3 latency with an increase in IQ in response to a passive oddball paradigm in their adult ID patients (Ikeda et al., 2009). Consistently with St. Clair and colleagues' findings (St. Clair et al., 1987), a prolonged auditory P3 latency has been observed in DS (Seidl et al., 1997; César et al., 2010). Additionally, patients with DS showed no P3 habituation to repeated stimulus presentation (Seidl et al., 1997) and a lower P3 amplitude (César et al., 2010). In contrast, Henderson et al. (2000) did not find differences between children with phenylketonuria and healthy controls in visual P3 latency and amplitude in an active oddball paradigm. The authors explained this absence of differences by appeal to the good dietary phenylalanine control of the patients, which limits the severity of ID, as well as the simplicity of the task. It may also arise from modality differences in the impairments found in ID, as suggested by the modality differences found by Van der Molen's group in FXS (Van der Molen et al., 2012b). Regarding autism, Bomba and Pang summarized the most common auditory P3 findings, indicating an unaffected latency and an attenuation in amplitude (Bomba and Pang, 2004). Thus, the P3 alterations found in FXS largely fit into general P3 latency prolongation in ID and amplitude reduction in autism.

### Associations between alterations in early stages of information processing and later stages of stimulus categorization in FXS

The observed P3 alterations in FXS have been explained by St. Clair and colleagues through a general malformation of limbic and associated medial temporal regions of the brain, in which P3 generators are assumed to be located (Smith et al., 1986). Part of this general malformation would be abnormal pyramidal neuronal functioning (Opitz et al., 1984). On the synaptic level, initial impairment in early stimulus processing, as reflected in N1 and P2 deviations, is likely to impair the formation of stimulus memory that is needed for the later N2, MMN, and P3 components. Thus, it is in line with these deviations that P3 latency is prolonged in FXS, since the latency of P3 is believed to reflect the duration of stimulus evaluation (Donchin, 1981). This assumed relation between the underlying synaptic impairments in the early stages of information processing, reflected through N1 and P2 enhancement, which compromises pre-attentive change detection (MMN) and stimulus categorization (N2), involuntary triggering of attention (P3a), and context updating (P3b), may also account for the deviations found in P3 amplitude. If the building of a memory trace for a stimulus was impaired, as suggested by the findings for the ERP components discussed so far, the stimulus categorization would be more difficult. This would have an influence on P3 latency and amplitude (Luck, 1998). Van der Molen and colleagues calculated the correlation between early sensory change detection components (N1 and P2) and active attentional components (N2b and P3) and found these to be positively associated in the auditory paradigms in controls, but not in FXS (Van der Molen et al., 2012b). Since there is no direct association between N1-P2 and N2b-P3, it seems probable that additional factors contribute to P3 alterations in FXS, despite the alterations found in previous components. Surprisingly, no direct association between MMN and P3a was found (Van der Molen et al., 2012a), even though it would seem plausible that pre-attentive change detection reflected by the MMN would have an effect on the mechanism detecting unattended stimuli, the cognitive process reflected by P3a. The authors contended that the missing association results from differences in the neuronal mechanisms generating the MMN and P3a (Van der Molen et al., 2012a). The MMN is believed to be generated in auditory and frontal cortices, and the P3a through frontocentral neuronal mechanisms, which also reflects assumed bottom-up (MMN) vs. top-down (P3a) information processing (Escera and Corral, 2007). Thus, the MMN activity is not a prerequisite for P3a generation. Moreover the two components are differently affected by changes in stimulus characteristics and contextual demands (Sussman, 2007; Wetzel and Schroger, 2007).

### Associations between P3b and behavioral performance in FXS

Even though only correlative and thus not necessarily causative, the association between P3b and behavioral performance measures found by Van der Molen and his group suggests that the impairments reflected by deviances in P3b characteristics may underlie the deficits in behavioral performance found in FXS (Van der Molen et al., 2012b). This is an important notion for the significance of ERP measures, since they reflect underlying neural mechanisms on the one hand and enable prediction of behavioral outcomes on the other. The differences between auditory and visual P3b amplitudes in FXS also manifested themselves in the behavioral results, since FXS patients performed significantly worse in the auditory than the visual oddball task (Van der Molen et al., 2012b). The authors discussed the possibility of a difference in the meaning of the stimuli, which might elicit more attention in the visual task (smiley faces vs. pure tones). However, this is not reflected in reaction times, which do not differ between the two tasks. Thus, Van der Molen and colleagues saw the explanation in poor auditory discrimination abilities rather than in poor task engagement (Van der Molen et al., 2012b). This is supported by findings indicating modality differences in FXS performance impairments (Sullivan et al., 2007; Van der Molen et al., 2010) and fits with the FXS modality differences found in the P2 and P3b components (Van der Molen et al., 2012b). Therefore, it can be assumed that the severity of stimulus processing impairments found in FXS varies across modalities, with the auditory modality being more affected than the visual modality. The authors matched lateralization differences found for P3a in FXS (Van der Molen et al., 2012a) to similar left lateralized brain activity during working memory tasks observed through neuroimaging studies (Hoeft et al., 2007). This could be interpreted as compensatory brain activity required for recruitment of attentional resources (Van der Molen et al., 2012a).

## General discussion and conclusion

### ERP alterations found in FXS: common in ID vs. specific for FXS

The ERP findings obtained in the five studies discussed above and summarized in Table 5, show that ERP is a useful measure to investigate impaired mechanisms of information processing in FXS, since several components showed a different profile in FXS patients compared to healthy controls.

**Table 5:** **Main ERP component findings in FXS patients compared with healthy controls**.

**Component**	**Latency**	**Amplitude**
**N1**	No difference (St. Clair et al., 1987; Rojas et al., 2001; Castrèn et al., 2003; Van der Molen et al., 2012a,b)	Increased (St. Clair et al., 1987; Rojas et al., 2001; Castrèn et al., 2003 Van der Molen et al., 2012a,b)
		No habituation (Castrèn et al., 2003; Van der Molen et al., 2012a)
**P2**	No difference (St. Clair et al., 1987; Van der Molen et al., 2012a,b)	Inconsistent
		*Increased* (St. Clair et al., 1987; Van der Molen et al., 2012a)
		*No difference* (Van der Molen et al., 2012b)	
**MMN**	No difference, Trend: prolonged, n.s. (Van der Molen et al., 2012a)	Decreased (Van der Molen et al., 2012a)
**N2**	Prolonged (St. Clair et al., 1987; Van der Molen et al., 2012a,b)	Inconsistent
		*No difference* (St. Clair et al., 1987)
		*Increased* (Van der Molen et al., 2012a,b)
**P3**	Prolonged (St. Clair et al., 1987; Van der Molen et al., 2012a,b)	Decreased (St. Clair et al., 1987; Van der Molen et al., 2012a,b)

However, reported results were not always consistent, especially in N2, for which two groups found enhanced amplitudes and one group reduced amplitudes, which might have been due to differences in study design. However, comparisons with studies investigating the development of ERPs with age suggest that some of the alterations might be caused by a general delay of brain maturation. According to the findings presented in this review, this could particularly concern MMN, N2, and P3. Further, some of the alterations might be common in clinical populations sharing symptoms with FXS, like other forms of ID or autism. Indications for general alterations in ID are found for N1 habituation, MMN amplitude, N2 and P3 latency and P3 amplitude. In contrast to that, N1 and P2 amplitude alterations seem more FXS specific. It is possible that distinct syndrome-specific perturbations in early sensory processes influence later components in similar ways. To address this topic, it would be advisable to consider supplementary control groups, matching the FXS patients' stage of mental development. This could be done using either patients with other forms of ID or chronologically younger healthy controls. Furthermore, it would be interesting to study different age groups with FXS to investigate the developmental course of ERP components in FXS.

### Cascade of impaired neuronal mechanisms as a basis for symptoms in FXS

ERP results obtained so far in FXS consistently show a cascade of impaired mechanisms in electrical summation necessary for basic stimulus processing, attentional processing, and memory formation. This is consistent with some of the symptoms found in FXS, as attentional problems might be explained through synaptic processes probably also underlying the ERP deviances. Further, the described cascade of impaired mechanisms could be the basis for other symptoms found in FXS. For example, hyperarousal, hyperactivity, and anxiety in FXS might be related to neural hyperreactivity in response to sensory stimuli. Moreover, the formation of a cerebral stimulus representation might be impaired through synaptic dysfunction. It is likely that this difficulty in memory formation affects further learning, which then results in cognitive deficits.

### Future directions

All in all, the ERP results fit with the symptoms found in FXS, as well as the anatomical findings obtained through brain imaging studies and assumptions concerning underlying neuronal mechanisms gained in animal models. Until now only a few FXS ERP studies have been published, so much remains to be discovered. Since existing ERP studies mainly focused on the auditory modality, other modalities should be investigated. The results obtained so far suggest that processing impairments vary across modalities. Moreover, deficits in the domain of social cognition could be addressed by using stimuli with more social relevance, like human voices or faces. It would be of particular interest to study other ERP components that are related to cognitive processes known to be impaired in FXS. For example, the face-specific N170 would be a promising candidate, since some evidence for impaired face recognition in FXS is reported (Turk and Cornish, 1998). Further, language-related ERPs like the N400, which occurs in response to violations of semantic expectations (Luck, 2005), or the P600, which is evoked by syntactic violations, would be interesting, since language is among the most impaired cognitive functions in FXS. Additionally, habituation of more ERP components besides N1 could be investigated. Finally, ERP studies might be helpful as outcome measures in clinical trials to assess the influence of medical treatment on the synaptic mechanisms reflected by ERP components.

### Conflict of interest statement

The authors declare that the research was conducted in the absence of any commercial or financial relationships that could be construed as a potential conflict of interest.

## Footnotes

^1^Some of the listed specifications for St. Clair's study population were detailed elsewhere (St. Clair and Blackwood, 1985; Primrose et al., 1986).
